# Association between schizophrenia and violence among Chinese female offenders

**DOI:** 10.1038/s41598-017-00975-2

**Published:** 2017-04-11

**Authors:** Jun Wang, Chun Li, Xiao-min Zhu, Si-mei Zhang, Jian-song Zhou, Qi-guang Li, Qun Wang, Shao-ling Zhong, Chee H. Ng, Gabor S. Ungvari, Yu-tao Xiang, Xiao-ping Wang

**Affiliations:** 1 Mental Health Institute of the Second Xiangya Hospital, Central South University. National Clinical Research Center on Mental Disorders & National Technology Institute on Mental Disorders. Hunan Key Laboratory of Psychiatry and Mental Health, Changsha, Hunan China; 2 Department of Psychiatry, University of Melbourne, Melbourne, Victoria Australia; 3 The University of Notre Dame Australia/Marian Centre, Perth, Australia; 4 School of Psychiatry & Clinical Neurosciences, University of Western Australia, Perth, Australia; 5 Faculty of Health Sciences, University of Macau, Macau SAR, China

## Abstract

Little is known about the association between schizophrenia and violence in women in China. This study aimed to examine the association between schizophrenia and violence in Chinese female offenders. Fifty-two schizophrenia patients were identified from the female offenders who received forensic psychiatric assessments in 2011 in Hunan province, China. Using a propensity score matching method, 104 matched controls without psychiatric disorders were selected from female criminals in Hunan province. Violent offences and homicides were verified and recorded. The percentages of violent offences and homicides were significantly higher in female offenders with schizophrenia than in controls (78.8% vs. 30.8%, *P* < 0.001; 44.2% vs. 18.3%, *P* = 0.001, respectively). Multivariate logistic regression analyses revealed that diagnosis of schizophrenia, younger age at first offence, living in rural area and a lower education level were independently and positively associated with violent offences, while having a diagnosis of schizophrenia and lower education level were associated with homicides. There appears to be an independent and positive association between schizophrenia and violent offence in Chinese female offenders. Effective preventive approaches on violence in female schizophrenia patients are warranted.

## Introduction

Schizophrenia (SCZ) is a severe psychiatric disorder with an estimated lifetime prevalence of around 1% worldwide. Many studies found that SCZ was associated with an increased risk of violent behavior^1–4^. In past decades violence in patients with SCZ has been widely examined, but most of these studies only focused on male patients possibly because women are less likely to commit violence than men^5, 6^. Although violent offence, specifically homicide, is not common in women^5–7^, convincing evidence showed that the gender gap is significantly narrowed when considering those with SCZ. For example, female perpetrators had higher prevalence of SCZ than males^8, 9^. Female patients with SCZ had increased risk for violence offence compared to the general population^2, 3, 10, 11^, with odds ratios (ORs) even higher than men^3, 12^.

Notably, previous studies on the association between SCZ and violence could be biased due to several factors. Substance abuse is an important risk factor for violence, and earlier results often included co-existing substance abuse^1, 4, 13^. In a systematic review Fazel *et al*. found that the risk of violence in people with substance abuse without psychosis was higher than those with psychosis alone, but similar to those with co-morbid substance abuse and psychosis^1^. Moreover, self-reported measures of violence in both SCZ patients and control groups^9, 14, 15^ are frequently unreliable because violent behaviors are often deemed as disgraceful and socially undesirable, therefore are subject to under-reporting^16^. In addition, the association between SCZ and violence is closely associated with sociocultural and economic factors^9, 17–19^. Previous findings were virtually all obtained in Western countries^3, 8–10, 13–15^, and therefore could not be generalized to different contexts. To date, there have been no studies examining violence in female patients with schizophrenia in China.

We set out to examine the association between SCZ and violence in Chinese female offenders. Due to the lack of agreement about the definitions of violence we focused, in our study, on violent offences as determined by law. After a person is arrested due to a violent offence, their personal information is documented and verified in the criminal record by the law enforcement agency. In addition, since homicide is the most severe manifestation of violence, we also analyzed homicide separately. We thus hypothesized that among female offenders there would be an independent and positive association between SCZ and violent offending including homicide.

## Methods

### Participants and study site

This was a cross-sectional study conducted in Hunan province, China. Hunan province is an intermediate-economically developed province located in central China (i.e., its Gross Domestic Product ranked the 10th out of the 32 provinces, municipalities and autonomous regions nationwide), with a total population of around 70 million.

To ensure pre-existing mental disorder prior to the offence (rather than developing a mental disorder after the crime), only female offenders who received forensic psychiatric assessments were recruited for the patient group. The other inclusion criteria were: (1) female criminal offenders diagnosed with SCZ based on a clinical diagnostic interview by two psychiatrists with more than 10 years clinical experience according to the 4^th^ edition of Diagnostic and Statistical Manual of Mental Disorders (DSM-IV)^20^, (2) fluency in Chinese and the ability to comprehend the interview questions, and (3) willingness to participate in the study. Exclusion criteria included a history of past or current substance abuse. The control group included female criminal offenders in the Hunan Women’s Prison without any history of psychiatric disorders. Exclusion criteria included those who had substance abuse, mental retardation and dementia, and those who did not comprehend the interview contents. The Hunan Women’s Prison is the only women’s prison in Hunan province that houses all adult female prisoners except for those adjudicated Not Criminal Responsible on Account of Mental Disorder (NCRMD)^21^.

A total of 581 criminal offenders received forensic psychiatric assessments in Hunan in 2011; 496 were diagnosed to have at least one psychiatric disorder according to the DSM-IV, of whom 54 female offenders had a diagnosis of SCZ. One was excluded due to alcohol abuse prior to the offence and another was excluded due to insufficient information and inability to provide written informed consent. Finally, 52 female offenders fulfilled the study criteria and formed the patient group (all were aged between 18 and 61 years when committing the offence for which they were accused).

**Figure 1:**
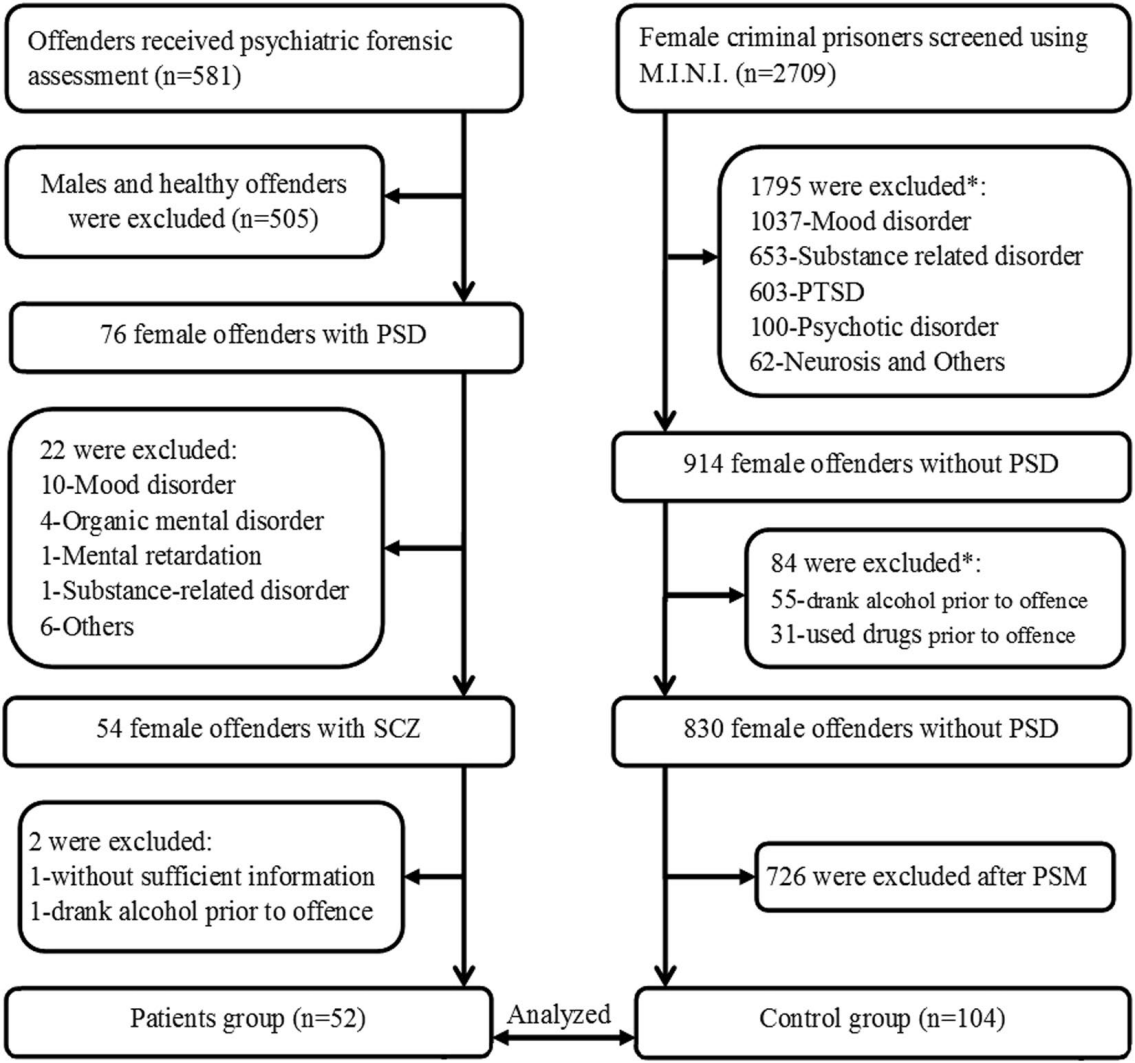
Flowchart of the recruitment process. PSD: psychiatric disorders; PTSD: posttraumatic stress disorder; PSM: propensity score matching; SCZ: schizophrenia; *Some subjects have more than one situations as listed below.

A total of 2,709 prisoners received mental health screening between December 2012 and November 2013; 914 (33.7%) did not meet any criteria of DSM-IV diagnosis, of whom 84 were excluded due to dependence on alcohol or (and) drugs prior to the offence. Finally, 830 female offenders met the study criteria for the control group. Since the control sample size was much larger than the patient group, and significant differences between the patients and controls were found in education level, place of residence, family history of psychiatric disorders and parental violent crime history, a propensity score matching analysis (PSM)^22^ approach was used to select the control participants. Finally, 104 subjects were selected for the control group (all were aged between 19 to 62 years when committing the offence for which they were serving their sentence) (Fig. 1).

### Assessment measures and evaluation

In Human province, prior to a forensic assessment the law enforcement agency needs to provide comprehensive relevant information, such as demographic information, criminal files and medical records of the offender. At least two senior forensic psychiatrists with over 10 years clinical experiences would conduct the forensic psychiatric assessment. Socio-demographic and clinical characteristics (year of birth, ethnicity, education level, marital status, place of residence, occupation and income before arrest, substance use, family history of psychiatric disorder and parental violent crime history, and criminal history (nature of offence, age at first and the most recent offence, and crime type) were collected by a standard data collection form designed for this study. Violent offence was defined as offences involving severe violent behavior, including homicide, serious assault, robbery, rape, sexual coercion and child molestation. Non-violent crime referred to other relatively moderate offences, such as larceny, fraud, smuggling, drug trafficking and extortion. Homicide was defined as convictions for murder, manslaughter and infanticide. For patients, the above data were extracted from their forensic case files and criminal files, and subsequently confirmed with their family members if available. For controls, information was self-reported and then confirmed with their criminal records and family members if available. Low income was defined as subject’s monthly income within the year before committing the offence lower than 1,000 Yuan (USD 148), which equals approximately to the average per capita income in Hunan in 2011.

The mental states of patients were evaluated in forensic psychiatric assessment centers by two psychiatrists according to DSM-IV. The mental health screening of the controls was conducted using the validated Chinese version of Mini-International Neuropsychiatric Interview (MINI, Chinese version 5.0.0)^23^. Prior to the screening, nine psychiatrist interviewers with at least 2 years of clinical experience undertook the MINI training at a 3-day workshop given by the Peking University Institute of Mental Health that had validated the MINI in China^23^. Prior to the commencement of the study, all interviewers attended an inter-rater reliability exercise on 10 clinically stable patients with different psychiatric disorders including schizophrenia, major depressive disorder, bipolar mania, generalized anxiety disorder, substance related disorder and posttraumatic stress disorder. The interviewers’ MINI diagnoses were compared with the best estimate clinical diagnoses. The kappa values for the interviewers were above 0.85, suggesting that the diagnostic agreement was acceptable.

### Ethical issue

The study was conducted in accord with the approved guidelines and regulations. The study protocol was approved by the Ethics Committees of the Second Xiangya Hospital, and the forensic psychiatric assessment centers, the Hunan Provincial Department of Justice and the Hunan Women’s Prison and Hunan Prison Administrative Bureau. All participants provided written informed consent.

### Statistical analyses

The comparisons between the patients and controls before and after PSM with regard to basic demographic and clinical characteristics were conducted separately using independent-samples *t-*test and Chi-squared test, as appropriate. Previous studies found that demographic variables including age, education level, economic condition, parental violent crime, employment and marital status were closely associated with violence offences^9, 17–19, 24^; therefore, these variables and those that significantly differed in the above univariate analyses were entered as covariates in the PSM^22, 25^. Univariate and stepwise multivariate logistic regression analyses were conducted to examine the independent correlates of violent offence and homicide. Violent offence or homicide was the dependent variable separately, while the variables that significantly differed in the above univariate analyses were entered as independent variables. All data analyses were conducted with the IBM SPSS version 22. The significance level was set at 0.05 (2-sided).

Given that the association between schizophrenia and violence in Chinese female offenders was the main parameter, the sample size was calculated based on the correlation coefficient (r) between schizophrenia and violence. As we could not locate any publications with similar study design, the sample size was calculated with an assumed medium effect size (r = 0.25), significance level = 0.05 (two sided) and power = 0.8 according to the Cohen’s power analysis method^26^. As a result, at least 123 participants would be needed. Since there were 52 female offenders in the patient group, the control groups were selected based on a 1:2 ratio in the PSM^22^. PSM is a statistical technique for causal inference in non-randomized studies that allows for conditioning on large sets of covariates^22, 25^. This technique was firstly reported in 1983, and the Rubin causal model was implemented for observational studies^27^. The logic behind the PSM is to achieve a balance across groups by matching each subject in a study group to one or more controls on propensity score^22^. The propensity score in the present study was calculated based on the aforementioned covariates, and the nearest neighbor matching algorithm was adopted to select controls. The PSM was performed with the SPSS following an established method^22^.

## Results

Univariate analyses revealed that the proportions of previous offence history, violent offences and homicides were consistently higher in SCZ patients than in controls in both pre- and post-PSM (all *P* < 0.05) (Table 1). The percentages of violent offences and homicides were significantly higher in female offenders with SCZ than controls (78.8% vs. 30.8%, *P* < 0.001; 44.2% vs. 18.3%, *P* = 0.001), respectively.

**Table 1:** Comparisons between patients and controls before and after propensity scores matching (n, %).

Variable	Before PSM			After PSM		
	Patients (n = 52)	Controls (n = 830)	*P*-value	Patients (n = 52)	Controls (n = 104)	*P*-value
***Demographic information***						
Han Chinese	47 (90.4)	739 (89.0)	0.762	47 (90.4)	88 (84.6)	0.320
Educational level						
Primary school and below	27 (51.9)	316 (38.1)	**0.047**	27 (51.9)	44 (42.3)	0.256
Junior middle school and above	25 (48.1)	514 (61.9)		25 (48.1)	60 (57.7)	
Unmarried	7 (13.5)	107 (12.9)	0.905	7 (13.5)	20 (19.2)	0.369
Unemployment	17 (32.7)	282 (34.0)	0.850	17 (32.7)	37 (35.6)	0.721
Low income	27 (51.9)	322 (38.8)	0.060	27 (51.9)	45 (43.3)	0.307
Living in rural area	40 (76.9)	293 (35.3)	**<0.001**	40 (76.9)	74 (71.2)	0.444
Family PSD history	14 (26.9)	38 (4.6)	**<0.001**	14 (26.9)	20 (19.2)	0.273
Parental violent crime	0 (0.0)	87 (10.5)	**0.014**	0 (0.0)	0 (0.0)	>0.99
***Criminological information***						
Offence history	23 (44.2)	39 (4.7)	**<0.001**	23 (44.2)	28 (26.9)	**0.030**
Age at first offence (years)	36.7 ± 11.8	36.8 ± 10.1	0.978	36.7 ± 11.8	37.9 ± 10.6	0.534
Less than 30	16 (30.8)	204 (24.6)	0.317	16 (30.8)	26 (25.0)	0.444
30 and above	36 (69.2)	626 (75.4)		36 (69.2)	78 (75.0)	
Age at present offence (years)	39.6 ± 10.7	37.2 ± 10.1	0.095	39.6 ± 10.7	38.8 ± 10.6	0.645
Less than 30	10 (19.2)	197 (23.7)	0.457	10 (19.2)	24 (23.1)	0.583
30 and above	42 (80.8)	633 (76.3)		42 (80.8)	80 (76.9)	
Violent Offence	41 (78.8)	254 (30.6)	**<0.001**	41 (78.8)	32 (30.8)	**<0.001**
Homicide	23 (44.2)	138 (16.6)	**<0.001**	23 (44.2)	19 (18.3)	**0.001**

PSD: psychiatric disorder; PSM: propensity score matching; ^#^Status before arrest; ^$^The age at present offence was used for persons without an offence history.

Of the 156 offenders (52 patients and 104 controls), 73 (46.8%) had violent offences and 42 (26.9%) had homicides. Diagnosis of SCZ, younger age at first offence, living in rural area and a lower education level were significantly associated with violent offences in both uni- and multivariate analyses (Table 2). Having a diagnosis of SCZ and lower education level were significantly and positively associated with homicide (Table 3).

**Table 2:** Univariate and multivariate analyses for violent offence (n, %).

Characteristic	Offence type		Univariate analysis		Multivariate analysis	
	Violent (n = 73)	Non-violent (n = 83)	OR (95% CI)	*P*-value	OR (95% CI)	*P*-value
Diagnosis of Schizophrenia	41 (56.2)	11 (13.3)	8.4 (3.8~18.4)	**<0.001**	9.3 (3.9~22.0)	**<0.001**
Younger age at first offence	26 (35.6)	16 (19.3)	2.3 (1.1~4.8)	**0.023**	4.0 (1.6~10.1)	**0.003**
Living in rural area	61 (83.6)	53 (63.9)	2.9 (1.3~6.2)	**0.007**	3.6 (1.4~9.4)	**0.009**
Low level of education	41 (56.2)	30 (36.1)	2.3 (1.2~4.3)	**0.013**	2.4 (1.1~5.3)	**0.034**
Chinese Han	64 (87.7)	71 (85.5)	1.2 (0.5~3.0)	0.698	NS	
Unmarried	13 (17.8)	14 (16.9)	1.1 (0.5~2.5)	0.877	NS	
Unemployment	24 (32.9)	30 (36.1)	0.9 (0.4~1.7)	0.669	NS	
Low income	38 (52.1)	34 (41.0)	1.6 (0.8~3.0)	0.166	NS	
Family PSD history	16 (21.9)	18 (21.7)	1.0 (0.5~2.2)	0.972	NS	
Previous offence	24 (32.9)	27 (32.5)	1.0 (0.5~2.0)	0.963	NS	
Young age at present offence	21 (28.8)	13 (15.7)	2.2 (1.0~4.7)	0.051	NS	

Bolded values: <0.05; PSD: psychiatric disorders; PSM: propensity score matching; NS: not significant in the final model.

**Table 3:** Univariate and multivariate analyses for homicide (n, %).

Characteristic	Offence type		Univariate analysis		Multivariate analysis	
	Homicide (n = 42)	Non-homicide (n = 114)	OR (95% CI)	*P*-value	OR (95% CI)	*P*-value
Diagnosis of Schizophrenia	23 (54.8)	29 (25.4)	3.5 (1.7~7.4)	**0.001**	4.0 (1.8~8.9)	**0.001**
Low level of education	25 (59.5)	46 (40.4)	2.2 (1.1~4.5)	**0.035**	2.2 (1.0~4.7)	**0.044**
Living in rural area	35 (83.3)	79 (69.3)	2.2 (0.9~5.5)	0.085	NS	
Chinese Han	36 (85.7)	99 (86.8)	0.9 (0.3~2.5)	0.855	NS	
Unmarried	5 (11.9)	22 (19.3)	0.6 (0.2~1.6)	0.284	NS	
Unemployment	13 (31.0)	41 (36.0)	0.8 (0.4~1.7)	0.560	NS	
Low income	20 (47.6)	52 (45.6)	1.1 (0.5~2.2)	0.824	NS	
Family PSD history	10 (23.8)	24 (21.1)	1.2 (0.5~2.7)	0.712	NS	
Previous offence	11 (26.2)	40 (35.1)	0.7 (0.3~1.4)	0.295	NS	
Young age at first offence	11 (26.2)	31 (27.2)	1.0 (0.4~2.1)	0.900	NS	
Young age at present offence	11 (26.2)	23 (20.2)	1.4 (0.6~3.2)	0.421	NS	

Bolded values: <0.05; PSD: psychiatric disorders; PSM: propensity score matching; NS: not significant in the final model.

## Discussion

There were only a total of 581 criminal offenders who received assessments in Hunan in 2011. In China, according to relevant regulations, only the police, prosecutors, and judges are qualified to apply for a forensic psychiatry assessment for a criminal offender who is suspected to have a current or history of psychiatric disorder^28^. If the offender and/or his/her family wish to apply for a forensic psychiatric assessment, the application requires prior approval by the local relevant law enforcement agency, such as prosecutors and court; hence this may partly explain the low assessment rate.

This is the first study examining the association of SCZ with violent offending and homicide in female offenders in China. We found that SCZ is significantly associated with an increased risk for violent offence and homicide in this female population, which supports our hypothesis. Further, of the 2,709 female prisoners, 1,795 (66.3%) had lifetime or current psychiatric disorder as assessed by the MINI. This figure in fact is lower than the findings in the US; Lynch *et al*. examined nine jails and found that 91% of participants met lifetime criteria and 70% met 12-month or current criteria for at least one psychiatric disorder as measured by the Composite International Diagnostic Interview (CIDI)^29^.

Several large-scale epidemiological surveys concerning violent offence and psychiatric disorders have been conducted in other countries^2, 3, 8, 9, 14, 15, 17^. However, the main limitations included the probable underestimation of prevalence of violence due to self- or family-report measures^9, 14, 15^, and the inadequate information on socioeconomic status^9^. In China, due to logistic reasons and inadequately trained interviewers, it is impossible to carry out a nationwide survey. Instead, well organized regional surveys are feasible. According to the Chinese *Criminal Code*, any offender should bear criminal responsibility if they have the ability to recognize and/or control his/her own conduct when committing the offence^21^. Accordingly, offenders suspected of having psychiatric disorders are mandated to receive a forensic psychiatric assessment. In the present study, we screened all persons who received forensic psychiatric assessments in Hunan province in 2011; therefore our sample is likely to represent all female offenders with a diagnosis of SCZ during this period. Similarly, the controls were selected in the same province, and thus were comparable to SCZ subjects in sociocultural and economic contexts. Moreover, the demographic characteristics and sample size of the two groups were matched using the PSM approach.

The reasons for the association between elevated risk of violence and SCZ are still unclear. Violence in SCZ could partly be attributable to psychotic symptoms, particularly delusions and hallucination^30^. For example, Nolan *et al*. found that approximately 20% of assaults in psychiatric inpatients were directly related to positive psychotic symptoms^31^. Some studies found the association between SCZ and violent offence was significantly mediated by comorbid substance abuse^1, 13^. In this study, however, independent association between SCZ and violence remained significant after excluding comorbid substance abuse, thus strengthening association between severe psychiatric disorders and violence that is beyond substance use^4^.

Apart from the diagnosis of SCZ, multiple logistic regression analysis revealed that younger age at first crime, living in rural area and lower education level were independently and positively associated with violent offences; in addition, lower education level is a risk factor for homicides. This is consistent with the findings obtained in the general population^19, 32^. The association between young age and more frequent crime/violence has been widely reported in criminological studies^32^. Pechorro *et al*. found that younger age of crime onset was closely associated with psychopathic-traits^33^, but Males and Brown argued that the association of violence with young age was probably mediated by poverty amongst youth^32^. The close relationship between socioeconomic background and violence has been frequently reported across different areas and ethnic races^19^. Persons involved in severe violence were more likely to have low socioeconomic status, such as low-income and low education level^19^. The positive association between rural residence and violence may also be mediated by poverty, since low economic level and poor education are likely to affect rural populations in developing countries^34^. In China rural poverty remains a critical problem^35^ even though the country is undergoing rapid urbanization^36^.

In recent years a number of studies have been conducted on preventive measures of violence in SCZ^37–40^. Certain antipsychotics, such as clozapine and olanzapine, have anti-aggression effects^38, 40^. A recent study found that violent offences in psychiatric patients receiving antipsychotics decreased by 45% when compared to un-medicated periods^39^. In addition, poor treatment adherence was a contributing factor of violent offences^39^, therefore long-acting formulations may have a potential role in reducing violence in SCZ patients. Moreover, we found that lower education level is a risk factor of violence. Improving education levels as well as mental health literacy may be a potentially useful approach to lower the risk of violence in SCZ. Previous studies have also found that appropriate cognitive, behavioral and life skills training could reduce aggressive behavior in young male violent offenders^41, 42^.

Several limitations of this study should be addressed. First, relevant information, such as the overall number of violent assaults and the proportion committed by women during the same period in Human province, were not available for comparison in this study. In addition, IQ was not examined in the controls, although those who did not comprehend the interview contents were excluded from this study. Third, since this study was conducted only in Hunan province, the findings need to be replicated in other areas of China. Finally, as less severe forms of violence were not recorded in criminal files, the association between less severe violence and SCZ could not be examined.

In conclusion, the results of this study showed an independent and positive association between schizophrenia and violent offending in Chinese female offenders. The findings need to be replicated in future multicenter studies with larger sample sizes. Effective preventive approaches on violence in SCZ female patients are warranted.
